# Major hurdles of immune-checkpoint inhibitors in pancreatic ductal adenocarcinoma

**DOI:** 10.20517/cdr.2022.142

**Published:** 2023-05-30

**Authors:** Liia Akhuba, Zhanna Tigai, Dmitrii Shek

**Affiliations:** ^1^School of Health Sciences, Western Sydney University, Sydney, NSW 2145, Australia.; ^2^Accreditation Centre, RUDN University, Moscow 117198, Russia.; ^3^Blacktown Clinical School, Western Sydney University, Sydney, NSW 2145, Australia.; ^4^Blacktown Mt Druitt Hospital, Sydney, NSW 2145, Australia.; ^5^Westmead Institute for Medical Research, Sydney, NSW 2145, Australia.

**Keywords:** Pancreatic cancer, immune-checkpoint inhibitors, tumor resistance, microenvironment

## Abstract

In 2030, pancreatic ductal adenocarcinoma (PDAC) will become the second leading cause of cancer-related mortality in the world. Unfortunately, neither conventional chemotherapy nor novel immunotherapeutic strategies can provide durable responses and the survival prognosis remains very low. PDAC is notorious for its immune-resistant features and unique genomic landscape facilitating tumor escape from immunosurveillance. Novel immune-checkpoint inhibitors (ICI) failed to show promising efficacy and other multi-modal approaches are currently being validated in multiple clinical trials. In this paper, we provide our opinion on the major mechanisms responsible for PDAC resistance to ICI therapy and provide our view on future strategies which may overcome those barriers.

Pancreatic ductal adenocarcinoma (PDAC) represents a major challenge in modern oncology^[1]^. It is predicted that by 2030 PDAC will become the second leading cause of cancer-related death^[2]^. Surgery is curative at earlier stages, whereas advanced or metastatic stages are almost impossible to treat^[3]^. Conventional chemotherapy can only provide a short partial remission with 5-year overall survival (OS) of less than 9% in patients with advanced PDAC^[4]^. Recent discoveries in cancer immunology have led to the successful use of immune-checkpoint inhibitors (ICIs) in treating advanced solid malignancies. ICIs are monoclonal antibodies that target immune checkpoints such as cytotoxic T-lymphocyte associated antigen 4 (CTLA-4), programmed cell death protein 1 (PD-1) with its ligands PD-L1/L2 and other expressed by antigen-presenting cells (APCs) and T cells [Figure 1]^[5]^. ICIs have shifted treatment paradigms for melanoma, non-small cell lung cancer (NSCLC), and hepatocellular carcinoma^[6,7]^. Unfortunately, PDAC has shown incredible resistance to immunotherapy^[8]^. To date, US Food and Drug Administration (FDA) has only approved PD-1 inhibitor *pembrolizumab*, albeit only for patients with high microsatellite instability (MSI-H)^[9]^. Unfortunately, the majority of patients (~ 97%) with microsatellite stable status (MSS) are not benefited from ICIs and their outcomes remain critically poor^[10]^. Early trials combining chemotherapy with ICIs also fail to show any superior efficacy in MSS patients^[9]^. This paper provides an opinion on factors responsible for PDAC resistance to ICIs and potential strategies to overcome this issue.

**Figure 1:**
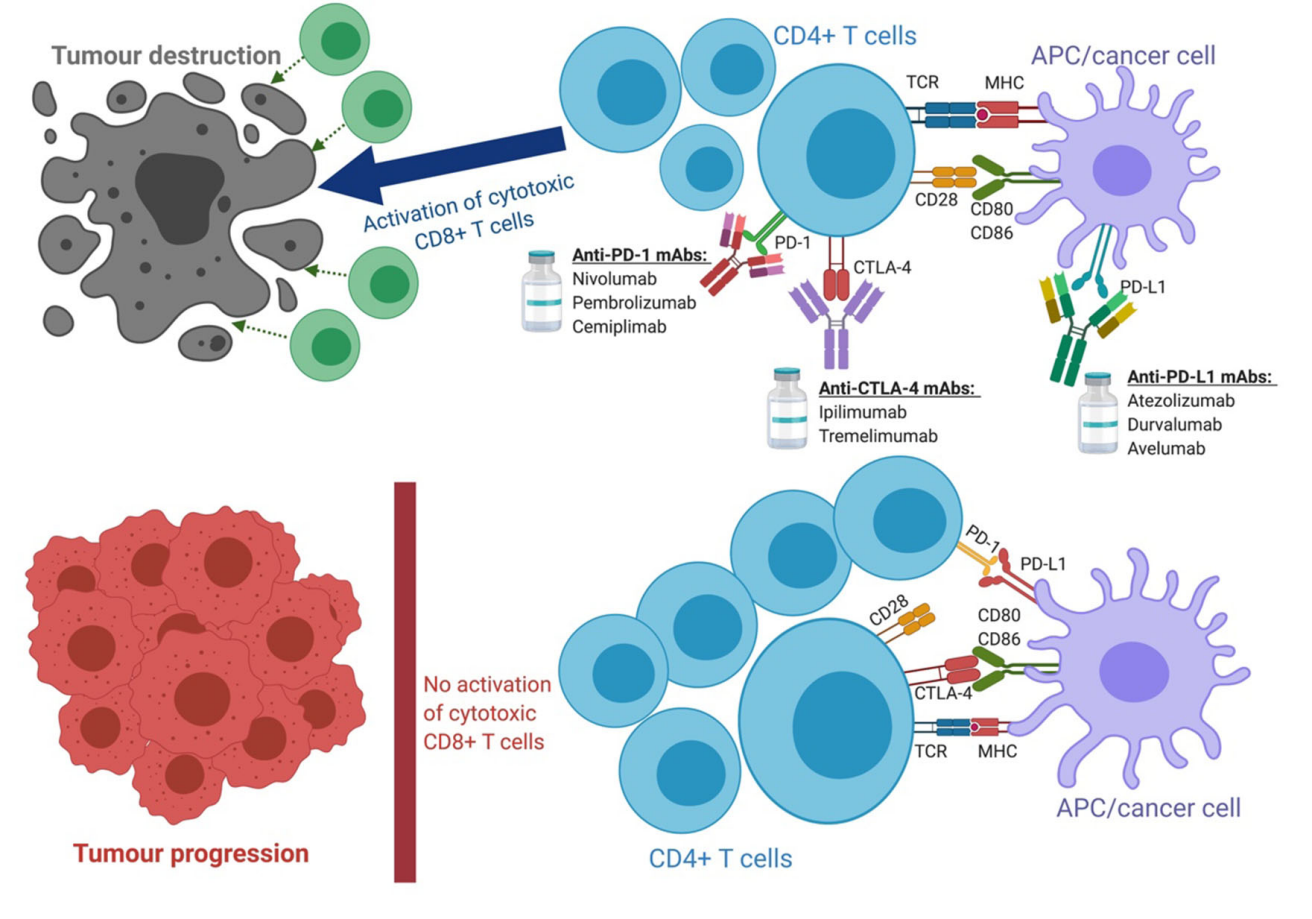
Mechanisms of immune-checkpoint inhibitors. ICIs target unique inhibitory checkpoint molecules expressed by T- and antigen-presenting cells. By blocking those receptors, ICIs promote the proper induction and differentiation of T cell-mediated immunity. In contrast, the absence of ICIs results in successful priming of checkpoint receptors with their ligands, thus inhibiting TCR activation overall, leading to cancer escape from immunosurveillance. APC: antigen-presenting cell; CD: cluster of differentiation; CTLA-4: cytotoxic T-lymphocyte associated antigen 4; mAb: monoclonal antibody; MHC: major histocompatibility complex; PD-1: programmed cell death protein 1; PD-L1: programmed cell death protein 1 ligand 1; TCR: T cell receptor.

Classically, PDAC has an immunologically “cold” tumor microenvironment^[11]^ characterized by abundant infiltration of myeloid cells and a small number of infiltrating T- and NK (natural killer) cells [Figure 2]. A few studies suggested that focal adhesion kinases (FAK) can regulate the fibrotic features of cold tumors, including the immunosuppressive microenvironment^[12,13]^. The data from *in vitro* studies on the synergistic efficacy of FAK + PD-1 inhibitors showed promising responses and resulted in further testing of this regimen in clinical trials. Other factors of resistance are low mutational burden and complex immunosuppressive features able to inhibit T cell priming and trafficking, resulting in lower efficacy of immunotherapy^[14]^.

**Figure 2:**
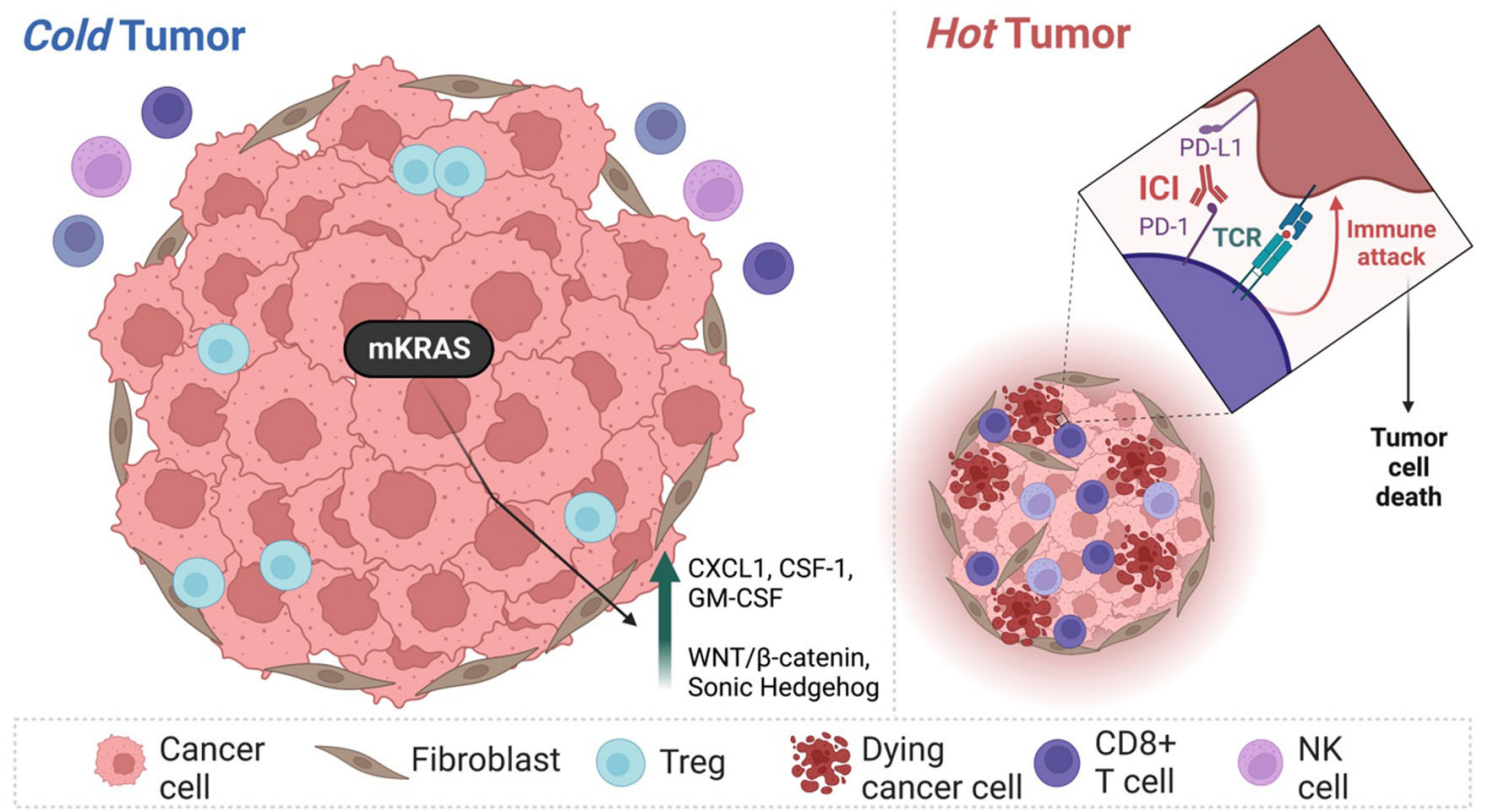
Mechanisms of pancreatic cancer resistance to immune-checkpoint inhibitor therapy. Pancreatic ductal adenocarcinoma is known as a tumor with a "cold" microenvironment characterized by a small number of CD8+ T- and NK cells, an abundance of regulatory T (immunosuppressive) cells, and poor response to ICI therapy. Mutation in *KRAS* gene (mKRAS) allows pancreatic cancer cells to induce expression of granulocyte-macrophage colony-stimulating factor (GM-CSF), chemokine C-X-C motif ligand 1 (CXCL1) and C-C motif chemokine ligand 4 (CCL4) playing a crucial role in immunosuppression. Moreover, mKRAS leads to upregulation of WNT/β -catenin pathway and Sonic Hedgehog pro-inflammatory pathways overall, inhibiting the ICI therapy.

Stromnes *et al*. reported that analysis of tumor samples revealed that PDAC has a lower number of effector T cells and lower clonality of T cell receptors as compared to other solid tumors that can be successfully managed by ICIs^[15]^. Genome studies have established that PDAC almost ubiquitously has activating *KRAS* (Kirsten ras oncogene) mutations^[16]^. Conventionally, mKRAS is known to be associated with tumor proliferation and metastasis; however, recent results of high-throughput studies have established that mKRAS may orchestrate downstream signaling responsible for immunosuppression^[17]^. A few *in vitro* studies established that mKRAS inhibits the expression of MHC-I, CD47, and PD-L1^[18,19]^. It is known that PD-L1 is a crucial marker for ICI efficacy in non-small cell lung cancer^[20]^. Perhaps the lower expression of checkpoint proteins (targets) negatively impacts ICI therapy and explains its lower effectiveness in PDAC patients. Secondly, mKRAS can upregulate the expression of GM-CSF and CXCL1, which are involved in the recruitment of myeloid-derived suppressor cells known for their immunosuppressive features^[21,22]^. Furthermore, mKRAS can downregulate the expression of CCL4 via WNT/β -catenin pathway^[23]^. CCL4 is an important factor for recruiting dendritic cells; major APCs require FOR priming T cell response and activating the cytotoxic cascade^[24,25]^. A lower number of APCs impacts the tumor escape from immunosurveillance. Additionally, mKRAS promotes signaling via the Sonic Hedgehog pathway and can induce expression of matrix metalloproteinase 7 (MMP-7)^[26]^ as well as selectively target lysosomal degradation of MHC-I molecules through an autophagy-dependent mechanism, thus negatively impacting ICI therapy^[19]^. Overall, it results in chronic inflammation and proliferation of the fibrotic stroma, thus complicating T cell trafficking^[27]^. The development of mKRAS-directed strategies may one day overcome this critical resistance mechanism and result in higher effectiveness of ICIs in PDAC.

In summary, PDAC is among the most immune-resistant tumors. Recent discoveries in understanding key elements of PDAC resistance to ICI therapy, including FAK^[28]^, mKRAS and other novel molecules^[29]^, have reshaped our view on future approaches for PDAC treatment. To effectively treat PDAC, it is crucial to elucidate the rational combinatorial approach(es) targeting both checkpoint proteins and non-redundant mechanisms of PDAC resistance, such as mKRAS. Moreover, novel therapeutic strategies should be selected based on patient’s individual genotype, which is responsible for high phenotypic heterogeneity observed across PDAC patients. Finally, mKRAS remains the bull’s eye for PDAC immunologic resistance; thus, the combinatorial approach of ICI + MEK (mitogen-activated protein kinase) inhibitors should be thoroughly studied in randomized trials. The synergistic effect of both drugs may improve clinical outcomes for PDAC patients in the near future.

## DECLARATIONS

### Copyright

© The Author(s) 2023.

## References

[1] Bray F, Ferlay J, Soerjomataram I, Siegel RL, Torre LA, Jemal A (2018). Global cancer statistics 2018: GLOBOCAN estimates of incidence and mortality worldwide for 36 cancers in 185 countries. CA Cancer J Clin.

[2] Rahib L, Smith BD, Aizenberg R, Rosenzweig AB, Fleshman JM, Matrisian LM (2014). Projecting cancer incidence and deaths to 2030: the unexpected burden of thyroid, liver, and pancreas cancers in the United States. Cancer Res.

[3] Von Hoff DD, Ervin T, Arena FP (2013). Increased survival in pancreatic cancer with nab-paclitaxel plus gemcitabine. N Engl J Med.

[4] Nevala-Plagemann C, Hidalgo M, Garrido-Laguna I (2020). From state-of-the-art treatments to novel therapies for advanced-stage pancreatic cancer. Nat Rev Clin Oncol.

[5] Johnson DB, Nebhan CA, Moslehi JJ, Balko JM (2022). Immune-checkpoint inhibitors: long-term implications of toxicity. Nat Rev Clin Oncol.

[6] Shek D, Akhuba L, Carlino MS (2021). Immune-checkpoint inhibitors for metastatic colorectal cancer: a systematic review of clinical outcomes. Cancers.

[7] Shek D, Read SA, Nagrial A (2021). Immune-checkpoint inhibitors for advanced hepatocellular carcinoma: a synopsis of response rates. Oncologist.

[8] Ho WJ, Jaffee EM, Zheng L (2020). The tumour microenvironment in pancreatic cancer - clinical challenges and opportunities. Nat Rev Clin Oncol.

[9] Akhuba L, Tigai Z, Shek D Where do we stand with immunotherapy for advanced pancreatic ductal adenocarcinoma: a synopsis of clinical outcomes. Biomedicines.

[10] O'Reilly EM, Oh DY, Dhani N (2019). Durvalumab with or without tremelimumab for patients with metastatic pancreatic ductal adenocarcinoma: a phase 2 randomized clinical trial. JAMA Oncol.

[11] Binnewies M, Roberts EW, Kersten K (2018). Understanding the tumor immune microenvironment (TIME) for effective therapy. Nat Med.

[12] Coppola S, Carnevale I, Danen EHJ (2017). A mechanopharmacology approach to overcome chemoresistance in pancreatic cancer. Drug Resist Updat.

[13] Jiang H, Hegde S, Knolhoff BL (2016). Targeting focal adhesion kinase renders pancreatic cancers responsive to checkpoint immunotherapy. Nat Med.

[14] Mueller S, Engleitner T, Maresch R (2018). Evolutionary routes and KRAS dosage define pancreatic cancer phenotypes. Nature.

[15] Stromnes IM, Schmitt TM, Hulbert A (2015). T cells engineered against a native antigen can surmount immunologic and physical barriers to treat pancreatic ductal adenocarcinoma. Cancer Cell.

[16] Biankin AV, Waddell N, Kassahn KS, Australian Pancreatic Cancer Genome Initiative (2012). Pancreatic cancer genomes reveal aberrations in axon guidance pathway genes. Nature.

[17] Collins MA, Bednar F, Zhang Y (2012). Oncogenic Kras is required for both the initiation and maintenance of pancreatic cancer in mice. J Clin Invest.

[18] El-Jawhari JJ, El-Sherbiny YM, Scott GB (2014). Blocking oncogenic RAS enhances tumour cell surface MHC class I expression but does not alter susceptibility to cytotoxic lymphocytes. Mol Immunol.

[19] Yamamoto K, Venida A, Yano J (2020). Autophagy promotes immune evasion of pancreatic cancer by degrading MHC-I. Nature.

[20] Rizvi NA, Hellmann MD, Snyder A (2015). Cancer immunology. Mutational landscape determines sensitivity to PD-1 blockade in non-small cell lung cancer. Science.

[21] Bayne LJ, Beatty GL, Jhala N (2012). Tumor-derived granulocyte-macrophage colony-stimulating factor regulates myeloid inflammation and T cell immunity in pancreatic cancer. Cancer Cell.

[22] Pylayeva-Gupta Y, Lee KE, Hajdu CH, Miller G, Bar-Sagi D (2012). Oncogenic Kras-induced GM-CSF production promotes the development of pancreatic neoplasia. Cancer Cell.

[23] Lemieux E, Cagnol S, Beaudry K, Carrier J, Rivard N (2015). Oncogenic KRAS signalling promotes the Wnt/β-catenin pathway through LRP6 in colorectal cancer. Oncogene.

[24] Spranger S, Bao R, Gajewski TF (2015). Melanoma-intrinsic β-catenin signalling prevents anti-tumour immunity. Nature.

[25] Zeng G, Germinaro M, Micsenyi A (2006). Aberrant Wnt/β-catenin signaling in pancreatic adenocarcinoma. Neoplasia.

[26] Olive KP, Jacobetz MA, Davidson CJ (2009). Inhibition of Hedgehog signaling enhances delivery of chemotherapy in a mouse model of pancreatic cancer. Science.

[27] Bear AS, Vonderheide RH, O'Hara MH (2020). Challenges and opportunities for pancreatic cancer immunotherapy. Cancer Cell.

[28] Zhang J, He DH, Zajac-Kaye M, Hochwald SN (2014). A small molecule FAK kinase inhibitor, GSK2256098, inhibits growth and survival of pancreatic ductal adenocarcinoma cells. Cell Cycle.

[29] Cascioferro S, Petri GL, Parrino B (2020). Imidazo[2,1-b] [1,3,4]thiadiazoles with antiproliferative activity against primary and gemcitabine-resistant pancreatic cancer cells. Eur J Med Chem.

